# Association of the C allele of rs479200 in the *EGLN1* gene with COVID-19 severity in Indian population: a novel finding

**DOI:** 10.1186/s40246-024-00572-1

**Published:** 2024-01-30

**Authors:** Renuka Harit, Sajal De, Piyoosh Kumar Singh, Deepika Kashyap, Manish Kumar, Dibakar Sahu, Chander Prakash Yadav, Mradul Mohan, Vineeta Singh, Ram Singh Tomar, Kailash C. Pandey, Kapil Vashisht

**Affiliations:** 1 ICMR-National Institute of Malaria Research, New Delhi, India; 2 Academy of Scientific and Innovative Research (AcSIR), Ghaziabad, India; 3 All India Institute of Medical Sciences, Raipur, Chhattisgarh India; 4 ICMR-National Institute of Cancer Prevention and Research, Noida, India

**Keywords:** *EGLN1* gene, COVID-19, Hypoxia, rs479200, rs516651, Host genetic factors, Risk factors

## Abstract

The present study investigated two single nucleotide polymorphisms (SNPs)—rs479200 and rs516651 in the host *EGLN1*/*PHD2* gene for their association with COVID-19 severity. A retrospective cohort of 158 COVID-19 patients from the Indian population (March 2020 to June 2021) was enrolled. Notably, the frequency of C allele (0.664) was twofold higher than T allele (0.336) in severe COVID-19 patients. Here, we report a novel finding that the C allele of rs479200 in the *EGLN1* gene imparts a high risk of severe COVID-19 (odds ratio—6.214 (1.84–20.99) *p* = 0.003; 9.421 (2.019–43.957) *p* = 0.004), in additive inheritance model (adjusted and unadjusted, respectively).

**Supplementary Information:**

The online version contains supplementary material available at 10.1186/s40246-024-00572-1.

## Introduction

Coronavirus disease 2019 (COVID-19) has subsided globally, which was caused by severe acute respiratory syndrome coronavirus-2 (SARS-CoV-2). The clinical disease symptoms vary drastically; while majority of infections remain asymptomatic or mild, severe complications may ensue with mortalities ranging from 2 to 7% [1]. The severe symptoms of COVID-19 pneumonia included acute respiratory distress symptoms (hypoxia, dyspnea, hypocapnia or hypercapnia) [2]. Some of the symptoms in COVID-19 hypoxia overlap with high-altitude pulmonary edema (HAPE) along with atypical features such as preserved lung compliance, intrapulmonary shunt and mild dyspnea; indicating loss of the homeostatic oxygen-sensing system which regulates oxygen uptake and systemic delivery. Single nucleotide polymorphisms (SNPs) in host genes have been shown to be crucial in disease risk and response to exposures, either from a pathogen or from the environment [3]. Some of the important risk factors for the host that had been attributed to severe symptoms of COVID-19 were age, sex, body mass index (BMI), preexisting comorbidities and ethnicity [4]. In particular, the SNPs in the host apolipoprotein E (*ApoE*) [5], angiotensin converting enzyme 1 (*ACE1*) [6], transmembrane serine protease 2 (*TMPRSS2*) [7], C-C chemokine receptor type 5 (*CCR5*) [8] and human leukocyte antigen (*HLA*) [9] loci have been linked to the susceptibility and/or severity of COVID-19 [10].

The *EGLN1* gene on chromosome 1 has been comprehensively studied in the context of HAPE hypoxia [11]. *EGLN1* translates to 2-oxoglutarate-dependent dioxygenase (2ODD) which regulates hypoxia-inducible factor (HIF-α) via prolyl hydroxylation. Under hypoxic conditions, the inactivation of 2ODD leads to increased HIF-α levels and subsequent dimerization with HIF-β to further activate hypoxia response elements (HRE) [12]. The hypoxia responses may include, but are not limited to the alterations in metabolic, cellular and systemic responses inside the cells.

The present study aimed to explore the role of *EGLN1* gene variants (rs479200 and rs516651) in COVID-19 severity in the Indian population. To the best of our knowledge, there are no reports on the association of the *EGLN1* gene and COVID-19 severity [13]. Thus, we report a novel finding that the C allele of the SNP rs479200 is overrepresented and has potential association in patients with severe COVID-19.

## Methods

### Sample collection and DNA extraction

A retrospective cohort of 158 adult patients (age ≥ 18 years) with RT-PCR confirmed COVID-19 infection during the two COVID-19 waves (2020 and 2021) in India, was studied. Enrolled patients were stratified into asymptomatic, mild, and severe based on the requirement of supplemental oxygen delivery devices to manage hypoxia during hospitalization. Asymptomatic COVID-19 patients had no symptoms and did not receive supplemental oxygen. Mild COVID-19 patients required supplemental oxygen by a low-flow system (i.e., nasal cannula, face mask). While, severe COVID-19 patients required supplemental oxygen through a high-flow system (non-rebreathing face mask, high flow nasal cannula) or ventilator (non-invasive or invasive) support for > 24 h. duration [14]. We received clotted blood samples from COVID-19 patients from the biorepository at ICMR-NIMR, Delhi, India and AIIMS, Raipur, India. The study was approved by the Institutional Ethics Committees (IECs), AIIMS, Raipur [1379/IEC-AIIMSRPR/2020] & ICMR-NIMR, Delhi [PHB/NIMR/EC/2020/145]. Written consent was obtained before sample collection from the patients. Nucleic acid extractions were performed at the COVID-19 testing facility at ICMR-NIMR, Delhi, using appropriate precautionary measures and personal protective equipment (PPE).

### Genotyping of rs479200 and rs516651 in the *EGLN1* gene

The *EGLN1* gene sequence was retrieved from the National Center for Biotechnology Information (NCBI) using Gene ID: 54583 for egl-9 family hypoxia-inducible factor 1 [*Homo sapiens* (human)]. The reference SNPs (rs479200 and rs516651) were identified from the dbSNP Reference SNP (rs) database at chromosomal positions- chr1:231408034 (GRCh38.p13) & chr1:231406910 (GRCh38.p14), respectively. For rs479200, a 367 bp segment in the *EGLN1* gene was amplified using the primers- forward primer- 5`*CTCCCAGCACATCTGTGAAT*3` and reverse primer- 5`*TCGGATGGAAAGGTGGTAAAG*3`. The restriction fragment length polymorphism (RFLP) analysis for rs479200 was accomplished by restriction enzyme *BsrGI*-HF [T/GTACA] (New England Biolabs, USA) [15]. Various genotypes of rs479200 were deduced- homozygous (TT & CC), heterozygous (TC) (Additional file 1: Fig. S1). For rs516651, TaqMan SNP genotyping assay (Assay-ID: C___2816320_10) was procured and genotyping analysis was performed to deduce the genotypes (CC, CT & TT), as per the manufacturer’s instructions. Fourteen samples could not be genotyped for rs516651 using TaqMan SNP genotyping assay, due to low concentrations of DNA or other technical limitations.

Statistical analyses were performed using the Statistical Package for the Social Sciences (SPSS) version 23 (IBM, USA) to estimate categorized genotypes, allele frequencies based on sex and age, and Hardy–Weinberg equilibrium (HWE). Logistic regression analysis was performed in univariate, and multivariate models; interaction analysis for both SNPs to test the association of demographic and genotype variables with increasing clinical severity was also performed.

## Results

### Clinical and demographic profiles of Indian COVID-19 patients

Additional file 1: Table S1 shows the demographic profiles of the patients included in the study, classified according to disease severity. In our study, the mean age of the total number of participants was 45.9 ± 18.3; however, the mean age of severe COVID-19 patients was 34.9 ± 15.6, contrary to the notion of prevalent severe complications in older patients. The mean age of COVID-19 patients in the asymptomatic and mild disease groups was 49.7 ± 17.9 and 54.3 ± 15.7, respectively. In addition, a preponderance of male COVID-19 patients was observed compared to females, across the clinical categories and in total number of participants, but it is unclear if this distribution is attributable to social or biological causes.

### Genotyping of rs479200 and rs516651 in the *EGLN1* gene

The allele frequencies of both SNPs were uniform across the categories, except for the severe category, where the frequency of the C allele of rs479200 was twice (C > T: 0.664 > 0.336) compared to that of the T allele (Additional file 1: Table S2). The data showed high heterozygosity across the clinical categories and in the total population for rs479200 alleles. In gender wise distribution, male patients depicted a high frequency of the C allele of rs479200. For rs516651, the T allele was present only in the heterozygous condition and a complete absence of the homozygous T allele was observed in studied COVID-19 from India. In bivariate Chi square analysis, no significant difference was observed across gender, but rs479200 showed a significant difference in clinical category (p value 0.010). Alleles in both SNPs displayed Hardy Weinberg equilibrium status across all categories and gender.

### Regression analysis

Regression (logistic) analysis was performed in univariate and multivariate models to test the association of demographic (age and sex) and genotype variables with increasing clinical severity. Mild and severe category patients were compared with the asymptomatic category patients, separately. In the adjusted (multivariate) model, the effects of all tested variables; gender, age and SNPs (rs479200 & rs516651) were tested together, whereas in the unadjusted (univariate) model each dependent variable was tested in isolation for their association with the outcome of mild and severe clinical severity. Table 1 depicts the association analysis data of rs479200 genotypes with clinical outcomes. No significant association was observed when comparing asymptomatic and mild COVID-19 patients. However, the asymptomatic vs severe category of patients displayed high odds ratios (6.214 (1.84–20.99) *p* = 0.003; 9.421 (2.019–43.957) *p* = 0.004) in the additive inheritance model (adjusted and unadjusted, respectively) with the CC genotype of rs479200. Similarly, high odds ratio of 6.024 (1.58–22.967) (*p* = 0.009); 3.956 (1.399–11.188) *p* = 0.01 was observed for the C allele in the dominant model for rs479200 in adjusted and unadjusted conditions, respectively. The minor T allele of rs516651 could not be tested in the recessive model due to nonavailability of homozygous genotypes. None of the tested variables showed any association with mild outcomes when compared with asymptomatic outcomes in the studied population. We also performed genotype interaction analysis of the studied alleles in asymptomatic vs severe COVID-19 categories (Additional file 1: Table S3), wherein we observed the CC and TT genotypes of rs516651 and rs479200 to be protective for COVID-19 (0.182 (0.51–0.647) *p* = 0.008).

**Table 1:** Association of rs479200 and rs516651 in the *EGLN1* gene with clinical and demographic characteristics (Regression (logistic) analysis)

			Wald	Sig	Odds Ratio	Confidence interval
*Asymptomatic versus mild*						
rs479200 T > C	Dominant (CC + CT vs TT)	Unadjusted	0.388	0.533	1.327	(0.545–3.23)
		Adjusted	0.111	0.739	1.172	(0.461–2.976)
	Recessive (CC vs CT + TT)	Unadjusted	0	0.992	0.995	(0.369–2.68)
		Adjusted	0.003	0.956	0.971	(0.349–2.707)
	Additive (TT vs CT; TT vs CC)	Unadjusted TT vs CT	0.438	0.508	1.375	(0.535–3.532)
		Unadjusted TT vs CC	0.113	0.737	1.219	(0.384–3.871)
		Adjusted TT vs CT	0.135	0.713	1.201	(0.451–3.197)
		Adjusted TT vs CC	0.023	0.88	1.098	(0.326–3.696)
rs516651 C > T	Dominant (CC vs CT + TT)	Unadjusted	0.076	0.783	1.238	(0.271–5.653)
		Adjusted	0.047	0.828	0.844	(0.183–3.899)
*Asymptomatic versus severe*						
rs479200 T > C	Dominant (CC + CT *vs* TT)	Unadjusted	6.723	0.01*	3.956	(1.399–11.188)
		Adjusted	6.915	0.009*	6.024	(1.58–22.967)
	Recessive (CC vs CT + TT)	Unadjusted	5.045	0.025*	2.862	(1.143–7.163)
		Adjusted	3.705	0.054	2.798	(0.981–7.98)
	Additive (TT vs CT; TT vs CC)	Unadjusted TT vs CT	3.733	0.053	2.952	(0.984–8.854)
		Unadjusted TT vs CC	8.653	0.003*	6.214	(1.84–20.99)
		Adjusted TT vs CT	4.893	0.027*	4.779	(1.195–19.108)
		Adjusted TT vs CC	8.145	0.004*	9.421	(2.019–43.957)
rs516651 C > T	Dominant (CC vs CT + TT)	Unadjusted	0.531	0.466	0.6	(0.152–2.369)
		Adjusted	1.305	0.253	0.42	(0.095–1.86)

*p value significant (0.05)

## Discussion

**Fig. 1:**
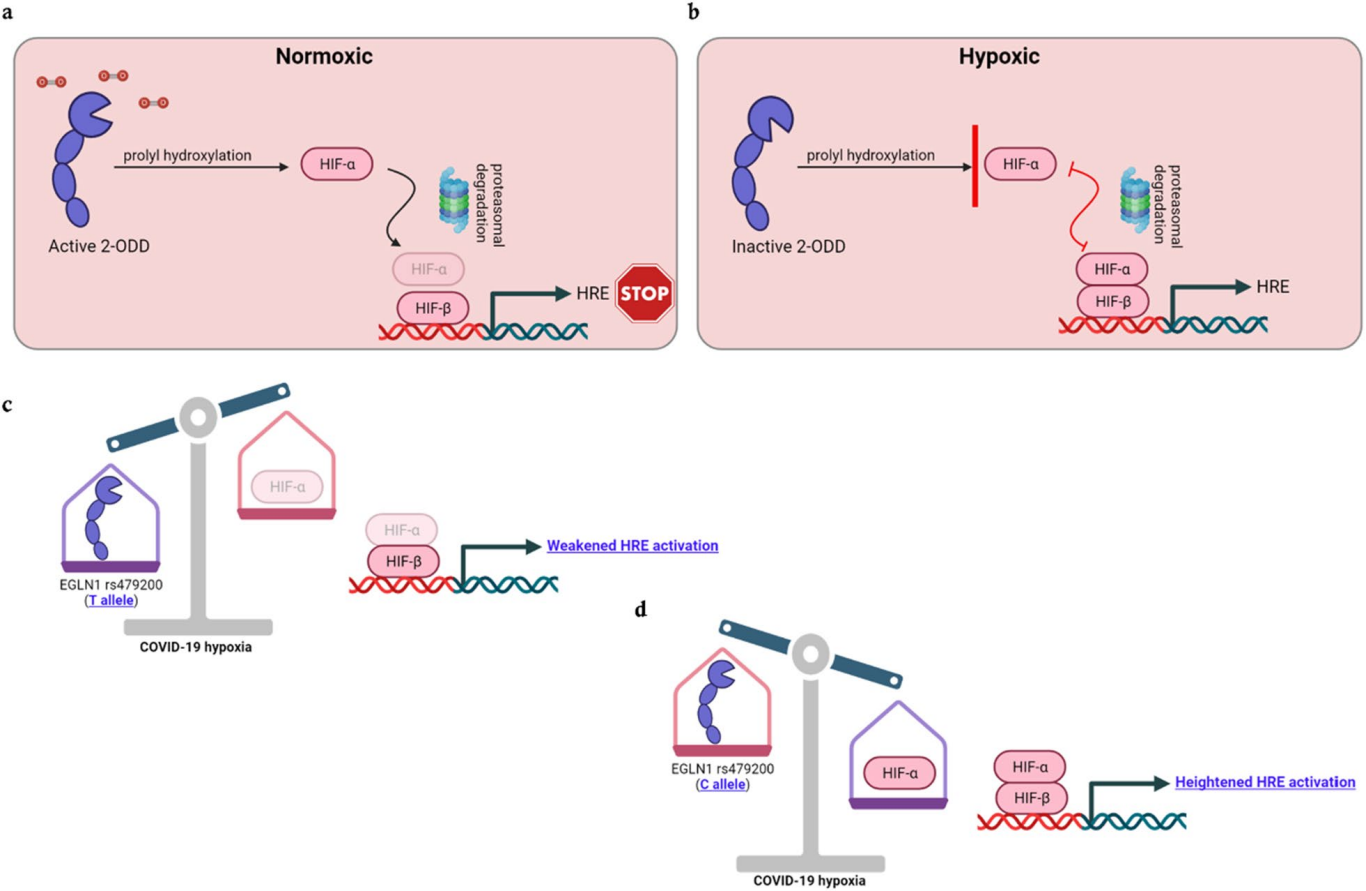
Schematic representation of the interplay between HIF-α and 2ODD (product of the *EGLN1* gene), inhibition of HRE activation under normoxic conditions (**a**) and activation of HRE via dimerization with HIF-β under hypoxic conditions (**b**). The postulated effect of the T-allele (**c**) in COVID-19 hypoxia weakening HRE activation and the effect of the C allele (**d**) in heightened HRE activation. (Created by BioRender trial version). (2ODD- 2-oxoglutarate-dependent dioxygenase; HIF-α- Hypoxia inducible factor- α; HIF-β- Hypoxia inducible factor- β; HRE- Hypoxic response elements.)

While there is a consensus that patients with severe COVID-19 present acute respiratory distress as in HAPE [2]; the similarities in the pathophysiology of severe COVID-19 and HAPE are debatable [16]. However, failure of oxygen sensing mechanisms is a characteristic feature in COVID-19 pneumonia [1]. To further correlate our observations with clinical findings, we postulated a hypothesis to describe how overrepresentation of the C allele in COVID-19 would lead to the progression of severe complications in response to COVID-19 hypoxia. Transcription of hypoxia response elements (HRE) in normoxic conditions is regulated and mediated by 2ODD- a gene product of the *EGLN1* gene; however, in hypoxic conditions, inactive 2ODD cannot inhibit HIF-α, and its dimerization with HIF-β leads to activation of HRE (Fig. 1a, b) Considering the genotype data of the *EGLN1* gene, the TT genotype of rs479200 is correlated with higher expression of the *EGLN1* gene resulting in overproduction of 2ODD and vice-versa [11]. Overproduction of 2ODD in the presence of the T allele (Fig. 1c) results in minimal HIF-α levels, rendering the individual’s hypo-responsive toward hypoxia. The C allele causes lower/inactive 2ODD causing the accumulation of high levels of HIF-α, and heightened hypoxic responses (Fig. 1d). The hyperresponsiveness to hypoxia with the presence of the C allele in severe COVID-19 might manifest in the form of pulmonary vascular alterations, overproduction of inflammatory cytokines, fluid accumulation in alveoli and cytokine storms.

Our preliminary findings with a limited sample size clearly demonstrated that the C allele is a potential risk factor for severe COVID-19 in Indian patients. However, a larger sample size and thorough statistical analysis are needed to validate the C allele as a prognostic biomarker for severe COVID-19 predisposition. It is important to note that due to the unprecedented lockdowns and lack of sufficient manpower, we were not able to record the detailed physiological parameters of the patients except for critical parameters available from the intensive care units (ICUs) of the hospitals. The availability of these parameters could have provided more insights into the role of the C allele in the severe category of COVID-19 patients. It is worth noting that deducing the genotype of SNP rs479200 can be easily accomplished in 4–6 h. time, equivalent to a COVID-19 RT-PCR test or quicker by using commercial Taqman assays.

## Conclusions

Our results clearly demonstrated an overrepresentation of the C allele of rs479200 in patients with severe COVID-19 from the Indian population and a novel association of the C allele with the severe category of COVID-19 was observed. The limitation of the study was the smaller sample size from Indian ethnicity; however, a major impetus of these findings can be realized with a retrospective analysis of the C allele in patients with severe COVID-19 with heterogeneous populations from different geographical locations. Therefore, the presence of the C allele as a risk factor in severe COVID-19 could serve as a feasible prognostic marker for risk assessment and prioritization of limited medical infrastructure.

### Supplementary Information

**Additional file 1**. Supplementary information.

## Data Availability

The data generated in the current manuscript are available upon request.
