# Correction: Association of the C allele of rs479200 in the *EGLN1* gene with COVID-19 severity in Indian population: a novel finding

**DOI:** 10.1186/s40246-024-00584-x

**Published:** 2024-02-08

**Authors:** Renuka Harit, Sajal De, Piyoosh Kumar Singh, Deepika Kashyap, Manish Kumar, Dibakar Sahu, Chander Prakash Yadav, Mradul Mohan, Vineeta Singh, Ram Singh Tomar, Kailash C. Pandey, Kapil Vashisht

**Affiliations:** 1 ICMR-National Institute of Malaria Research, New Delhi, India; 2 Academy of Scientific and Innovative Research (AcSIR), Ghaziabad, India; 3 All India Institute of Medical Sciences, Raipur, Chhattisgarh India; 4 ICMR-National Institute of Cancer Prevention and Research, Noida, India

**Correction: Human Genomics (2024) 18:7** 10.1186/s40246-024-00572-1

Following publication of the original article [1], the authors reported an error that the co-corresponding authorship of Dr. Kailash C Pandey has been inadvertently missed in the published version. The correct authorship has been updated in this Correction.

The original article [1] has been corrected.
