# Supplementary material for: Association of the C allele of rs479200 in the EGLN1 gene with COVID-19 severity in Indian population: a novel finding
Source: Hum Genomics. 2024 Jan 30;18:7. doi: 10.1186/s40246-024-00572-1 (PMC10826260; doi:10.1186/s40246-024-00572-1)
Supplement: Supplementary file 1 — Additional file 1. Supplementary information. [file 40246_2024_572_MOESM1_ESM.docx]

**Supplementary data**

**Table S1:** Clinical and demographic profiles of COVID-19 patients from India included in the current study.

|  | | **Asymptomatic** | | **Mild** | | **Severe** | | **Total** | |
| --- | --- | --- | --- | --- | --- | --- | --- | --- | --- |
|  |  | **N** | **%** | **N** | **%** | **N** | **%** | **N** | **%** |
| **Age**  **(Mean** ±**SD)** | | 49.7±17.9 | | 54.3±15.7 | | 34.9±15.6 | | 45.9±18.3 | |
| **Gender** | **Female** | 9 | 23.7% | 11 | 17.7% | 18 | 31.0% | 38 | 22.75 |
|  | **Male** | 29 | 76.3% | 51 | 82.3% | 40 | 69.0% | 120 | 71.86 |
|  | **Total** | **38** | **22.75** | **62** | **37.13** | **67** | **40.12** | **167** | **100.00** |

**Table S2:** Gender and clinical categories, genotypes and allele frequencies of rs479200 and rs516651 in the *EGLN1* gene.

| **rs479200** | **Gender** | | | **Classification** | | | **rs516651** | **Gender** | | **Classification** | | |
| --- | --- | --- | --- | --- | --- | --- | --- | --- | --- | --- | --- | --- |
|  | **Female** | | **Male** | **Asymp-**  **tomatic (A)** | **Mild (M)** | **Severe (S)** |  | **Female** | **Male** | **Asymp-tomatic (A)** | **Mild (M)** | **Severe (S)** |
| **TT** | **N** | 11 | 21 | 12 | 16 | 7 | **TT** | 0 | 0 | 0 | 0 | 0 |
|  | **%** | 28.20 | 17.10 | 31.60 | 25.80 | 10.40 |  | 0 | 0 | 0 | 0 | 0 |
| **TC** | **N** | 20 | 61 | 18 | 33 | 31 | **TC** | 3 | 15 | 3 | 5 | 10 |
|  | **%** | 51.30 | 49.60 | 47.40 | 53.20 | 46.30 |  | 9.1 | 16.3 | 10.3 | 12.5 | 16.1 |
| **CC** | **N** | 8 | 41 | 8 | 13 | 29 | **CC** | 30 | 77 | 26 | 35 | 52 |
|  | **%** | 20.50 | 33.30 | 21.10 | 21.00 | 43.30 |  | 90.9 | 83.7 | 89.7 | 87.5 | 83.9 |
| **Chi square**  **(p value)** | 3.483 (0.1752) | | | (A*M) 0.439 (0.803)  (A* S) **9.39 (0.009) *** | | | **Chi square**  **(p value)** | 1.025 (0.311) | | (A*M) 0.076 (0.783)  (A* S) 0.54 (0.462) | | |
| **T** | 0.538 | | 0.419 | 0.553 | 0.524 | 0.336 | **T** | 0.045 | 0.082 | 0.052 | 0.063 | 0.081 |
| **C** | 0.462 | | 0.581 | 0.447 | 0.476 | **0.664** | **C** | 0.945 | 0.919 | 0.949 | 0.938 | 0.920 |
| **HWE** | 0.039 | | 0.044 | 0.067 | 0.278 | 0.093 | **HWE** | 0.075 | 0.725 | 0.086 | 0.178 | 0.477 |
| **p value** | 0.843 | | 0.835 | 0.796 | 0.598 | 0.761 | **p value** | 0.784 | 0.395 | 0.769 | 0.673 | 0.490 |

(HWE- Hardy Weinberg Equilibrium)

**Table S3:** Genotype (SNP*SNP) interaction analysis of studied alleles with clinical categories

|  | **Wald** | **sig** | **Odds ratio** | **CI (95%)** |
| --- | --- | --- | --- | --- |
| **Asymptomatic Vs Mild** | | | | |
| TT (rs479200) * CC (rs516651) | 0.288 | 0.592 | 0.714 | (0.209-2.443) |
| T/C rs479200 ) * CC (rs516651) | 0.013 | 0.908 | 0.935 | (0.3-2.912) |
| **Asymptomatic Vs Severe** | | | | |
| TT (rs479200) * CC (rs516651) | 6.933 | 0.008 | 0.182 | (0.051-0.647) |
| T/C (rs479200) * CC (rs516651) | 1.144 | 0.285 | 0.57 | (0.204-1.596) |

**Figures**


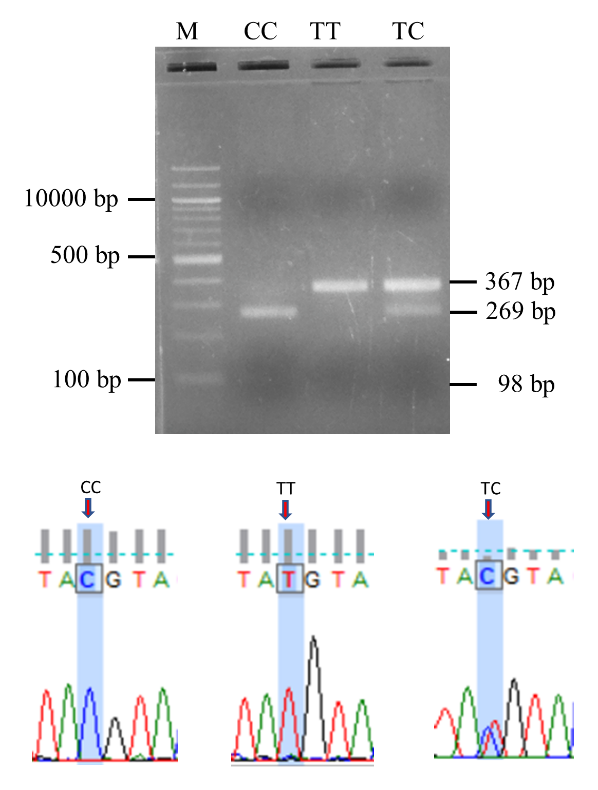


**Figure S1:** PCR-RFLP analysis of SNP rs479200 of *EGLN1* gene with enzyme *BsrGI* (T/GTACA).
